# Organizational transformation in health care: an activity theoretical analysis

**DOI:** 10.1108/JHOM-10-2018-0284

**Published:** 2019-08-08

**Authors:** Jeanette Kirk, Ove Andersen, Janne Petersen

**Affiliations:** 1 The Emergency Department, Clinical Research Centre, Amager and Hvidovre Hospital, University of Copenhagen, Hvidovre, Denmark; 2 Department of Public Health, University of Copenhagen, Copehagen, Denmark

**Keywords:** Culture, Implementation, Screening, Activity system analysis, Cross-continuum collaborations

## Abstract

**Purpose:**

Older patients are at high risk of hospital readmission, which has led to an increasing number of screening and intervention programs. Knowledge on implementing screening tools for preventing readmissions in emergency department (ED), where the primary focus is often the present-day flow of patients, is scant. The purpose of this paper is to explore whether a new screening tool for predicting readmissions and functional decline in medical patients>65 years of age could be implemented and its influence on cross-continuum collaborations between the primary and secondary sectors.

**Design/methodology/approach:**

The study took place in an ED in Denmark, in collaboration with the surrounding municipalities. An evaluation workshop with nurses and leaders from the ED and the surrounding municipalities took place with the aim of investigating the organizational changes that occurred in daily practice after the implementation of the screening tool. The workshop was designed and analyzed using cultural historical activity theory (CHAT).

**Findings:**

The results showed that it was possible to develop collaboration between the two sectors during the test period. However, the screening tool created different transformations for the municipality employees and in the ED. The contradictions indicated that the screening tool did not mediate a general and sustained transformation in the cross-continuum collaboration.

**Research limitations/implications:**

Screening tools are not objective, neutral or “acontexual” artifacts and must always be adapted to the local context and sectors. CHAT offers a perspective to understand the collective object when working with organizational transformations and implementation.

**Practical implications:**

The study have shown that screening tools are not objective, neutral or “acontexual” artifacts and must always be adapted to the local context. This is called adaption process. This adaption requires time and resources that should be taken into consideration from the beginning of introduction of new screens.

**Originality/value:**

This paper contributes with knowledge about CHAT which offers a way to understand the leading collective object when working with organizational transformations and implementation. CHAT focuses not only on the structural changes but also on the cultural aspects of organizational changes, which is important if we want to reach a sustained change and implement the new screening tool in different sectors.

## 1. Introduction

Patients aged 65 years and older are at increased risk of being admitted or readmitted to an emergency department (ED) (Samaras *et al.*, 2010). Older frail patients admitted to hospital are particularly at risk of functional decline and readmission (De Buyser *et al.*, 2014). This is a challenge socioeconomically, physically and psychologically for the patients and their relatives (Kamp and Hvid, 2012).

Structural changes to the National Hospital Plan were implemented in Denmark in 2007 with the main purpose to have one entry point for acute admissions through the ED, leading to fewer EDs (Andersen and Jensen, 2010). The consequence of these structural changes was that unselected patients with a variety of diseases and often with several diseases were admitted in the ED (Juul-Larsen *et al.*, 2017). Thus, ED personnel required increased awareness of the complexity of care in addition to focusing on acute and life-threatening symptoms. At the same time, the EDs became an ideal place to screen for at-risk patients, because almost all acute admissions came through the department. In total, 65 percent of these patients were discharged directly from the ED to the municipalities which, in Denmark, are responsible for home care (Hermansen *et al.*, 2015). These changes have led to the development of an increasing number of hospital- and community-based geriatric screening and intervention programs targeting older patients (Aminzadeh and Dalziel, 2002). However, research has shown that the staff members in EDs still prioritize acute treatment that can ensure patient flow, rather than basic nursing actions and screenings (Kirk and Nilsen, 2015). In addition, it is a challenge to implement screening tools targeted at older medical patients in EDs. The barriers include lack of resources, lack of understanding of screening tools compared with more sophisticated assessment tools and lack of adaptation to the local context (McCusker *et al.*, 2007; Meldon *et al.*, 2003). As a result, patients are not being screened in the ED, which might lead to less cooperation and communication between municipalities and hospitals, such that the municipality officials who take over responsibility for the patients get no information. Furthermore, the municipalities have no contact with nearly 50 percent of the patients, who are admitted or readmitted to the ED before their admittance, and these patients will therefore not be offered any kind of rehabilitation or home care when they are discharged (Koonkies *et al.*, 2015).

### 1.1 The Info-65 project

The Info-65 project was carried out from September 2013 to July 2014. The objectives of Info-65 were to explore whether a new screening tool for predicting readmission and functional decline in all medical patients >65 years of age acutely admitted to the ED can be implemented in daily clinical practice and whether it can predict readmission as well as develop cross-continuum collaborations between the hospital and the municipalities.

The tool consisted of three elements: routine blood tests for the presence of biomarkers (C-reactive protein and soluble urokinase-type plasminogen activator receptor) (Haupt *et al.*, 2012); three questions on: help at home, times out of the home and whether the patient has been hospitalized within the last six months; and examination of habitual gait speed with a 4-m walking test (Guralnik *et al.*, 2000).

In this paper, the aim is to explore whether a new screening tool for predicting readmissions and functional decline in medical patients>65 years of age could be implemented and its influence on the cross-continuum collaborations between the primary and secondary sectors.

In this study, implementation is understood to not only involve a change in practice but also build up the practitioners’ collaborative agency and motivation based on a new understanding of the idea of the activity and the collectively shared outcome (Engeström, 2000).

## 2. Methods

### 2.1 Setting

The study took place in a 600-bed university hospital in Copenhagen, Denmark, in collaboration with the surrounding municipalities and general practitioners. In Denmark, the public health care system is funded by the tax payers and provides free treatment for primary medical care, hospitals and home care services for all citizens. The Danish health care system is based on the Danish Health Act, 2005, which regulates who is responsible for the treatment, prevention and health promotion for Danish citizens. Whereas the regions are responsible for treatment within hospitals, including outpatient visits at the hospitals, the municipalities are responsible for other health activities, e.g. rehabilitation (Ministry of Health, 2005). The Health Act aims to ensure that visitation services are available to citizens after discharge from hospital. Part of this Health Act is a communication agreement, which aims to ensure cross-continuum communication of known citizens (Buch *et al.*, 2014). Known citizens are citizens who receive home care or whose health has deteriorated such that they have a new need for care from the municipalities. Accordingly, the municipalities will not receive information on citizens who are not already known to them after discharge from hospital.

### 2.2 Participants

The participants in Info-65 were nurses and leaders from Municipality B; a project nurse, general practitioners and leaders from Municipality K; a doctor, one nurse, two physiotherapists and their leader from the geriatric team (G) in the ED. Also, the research team participated in the workshop.

From September 1, 2013 to July 1, 2014, 3,666 patients from the two municipalities were acutely admitted and participated in the study; 1,506 (41 percent) of those were screened by the geriatric team in the ED. Patients were screened within 24 h after admission in the ED. If the patients were found to be at risk of readmission or functional decline, the municipality and the general practitioner were contacted and further supporting interventions were initiated in the municipalities. In Municipality B, the at-risk patients were offered three preventive home visits by a nurse. In Municipality K, the at-risk patients were offered three preventive home visits and data on the at-risk patients were sent to the general practitioner.

### 2.3 Cultural historical activity theory (CHAT)

Data were collected and analyzed using CHAT (Engeström, 1987). This theory emphasizes the importance of good theoretical understanding of how intervention causes change; individuals are seen as co-producers of societal and cultural development rather than as producers of their own development (Engeström, 2000). In health, understanding change requires theoretical insights that go beyond individual actors to viewing health care practice as a collective activity comprising multiple actors and stakeholders who must work collaboratively in the implementation process (Engeström and Sannino, 2010). CHAT also provides a set of instruments for innovating through expansive learning (Engeström, 1987), which cuts across individual and organizational learning.

The central insight in CHAT is that human interaction with the environment mediated by tools and signs leads to a specifically human form of activity and a principle of development that is based on cultural learning (Virkkunen and Kuutti, 2000). CHAT also highlights the historical evolution of human activity to understand why things have become as they are. In this study, we selected three theoretical concepts: objects, activity systems and contradictions. The three theoretical concepts are chosen because they offer a perspective for analyzing interactions between health care professionals and how these interactions influence the possibility for developing a collectively shared outcome. Drawing on CHAT, objects collaboration builds on collective objects (Engeström, 1987). The object of activity is understood as a focal entity or a desired outcome and consists of a concrete product or service that is being produced, e.g. a constant focus of prioritizing acute treatment that ensures the patient flow, rather than focusing on basic nursing actions and screenings (Kirk and Nilsen, 2015). Objects form the meaning and shared motive for the collective activity. They define and separate activities from each other. An entity becomes an object of human activity when its transformation is seen to meet a societal need and is invested with the meaning and motivating power related to meet that need (Engeström, 2008).

**Figure 1:**
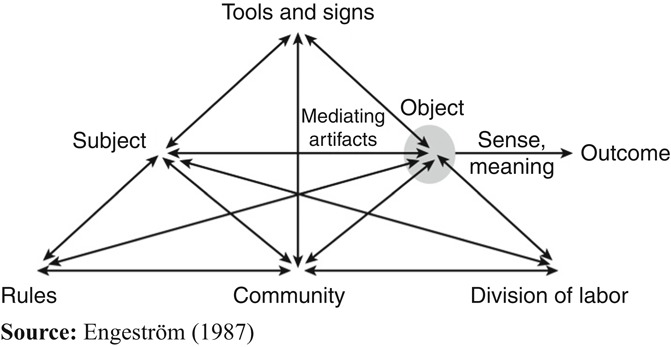
A complex model of an activity system

An activity system is a collective formation that has a complex mediational structure (Engeström and Sannino, 2010). Human activities are understood as a cultural system (Figure 1) mediated by intellectual and practical tools and signs used in the activity, and its rules and division of labor mediate the subject’s interaction with the object of the activity and with the other members of the community working on the object (Engeström, 1987).

Contradictions within and between activity systems are key to understanding the sources of problems, the innovative and developmental potential and transformations of activity (Engeström, 2000). Contradiction means that two things or processes that determine the practitioners’ actions pull the action in opposite directions. Primary contradiction occurs within all elements of the activity system (Engeström, 1990). When major changes take place in society, e.g. the patients’ hospital stay is shortened due to efficiency improvements or new treatments, such changes transform the primary contradiction into a secondary contradiction between some elements of the activity systems (Virkkunen and Kuutti, 2000). Overcoming these contradictions is possible through a reconceptualization of the object and motive for the activity and creation of a new tool. Tertiary contradictions occur in the activity system between the new tool and the existing form when implementing the new tool, and eventually quaternary contradictions emerge between the new activity and other activities from which it is functionally dependent.

To explore whether the new screening tool supports and develops cross-continuum collaborations, we use Engeström’s (2008) four modes of collaborations: coordination is a normal, scripted flow of interactions; disturbance in coordination takes place either when the object and the role of the actors clash or when there are competing scripts; cooperation is a mode of interaction in which the actors, instead of focusing on their respective individual objects and acting according to their respective roles, focus on a shared problem trying to find an acceptable way of solving it, and in solving the problem, they do not pay attention to the script; and communication is a mode of collaboration in which the actors reformulate their roles and the script of their interaction in relation to a sustained shared object. Transition from one mode to another takes place because of disturbances in the interaction. The four modes of collaborations build on the concept of a script. According to Virkkunen and Newnham (2013, p. 88), a script “is a habitual, tacitly expected order of interacting participants’ actions and the operations through which the action are carried out.”

## 3. Design

### 3.1 Ethnographic field study

The first step in activity theoretical analysis is to gain an insight into the problems and get a grasp of the need state and primary contradiction beneath the surface of the problems, doubts and uncertainties experienced among the participants of the activity. We delimited the activity system by performing a three-month ethnographic field study with participants’ observations in the ED (Spradley, 1980). The field study and the cultural analysis showed that a flow culture existed in the ED, understood as an activity system whereby the practitioners collectively tried to achieve the object: to secure a free bed and maintain a constant flow of patients (Kirk and Nilsen, 2015). A flow culture leads to a strong focus on securing vacant beds, which impeded the nurses’ use of evidence-based screenings tools and guidelines in clinical practice.

### 3.2 Contradiction study

The field study showed a mixed picture in relation to the inclusion and exclusion of the nurses’ use of screening tools and guidelines (Kirk and Nilsen, 2016). As we wanted to introduce a new screening procedure in the same ED, it became relevant to understand these inclusion and exclusion processes that occurred in daily practice. So, we conducted a contradiction analysis (Yagamata-Lynch, 2010). The contradiction analysis showed that screenings and guidelines that did not support the collective activity were perceived as flow stoppers. A flow stopper is an action that, despite execution, has no influence on how quickly the patients go through the department. Such an action became a flow stopper because the nurses spent time on it with the risk of losing overview and thereby increasing the risk that the flow could not be maintained (Kirk and Nilsen, 2016). These two studies showed that a flow culture among the health professionals in the ED may be an important barrier to the use of the new screening tool if it does not support the flow of patients.

### 3.3 Barrier screening

To establish who should test and implement the screening, the nurses or the geriatric team, we performed barrier screening (Cane *et al.*, 2012; Michie, 2005). The aim of the barrier screening was to explore and understand the perceived barriers and facilitators from the perspectives of the nurses and the geriatric team in relation to implementation of the new screening tool before testing the tool. Geriatric assessments have been found to increase patients’ chances of survival and the opportunity to remain in their own home (Ellis *et al.*, 2011), which suggests that the geriatric team should handle the new screening. On the other hand, the cultural analysis showed screening patients for, e.g. Early Warning Score, was an integral part of the everyday tasks for the ED nurses (Kirk and Nilsen, 2015). This suggests that the nurses should handle the new screening. The barrier screening (Kirk *et al.*, 2016) showed that the geriatric team perceived themselves as experts on older medical patients and had a different approach to the patients than the ED nurses. Therefore, the new screening tool better suited their professional identity, and it was decided that they should perform the screening.

### 3.4 Workshop

To evaluate the Info-65 project, we held a final workshop where the results were presented. We designed the workshop based on CHAT. The workshop method helped us to generate knowledge about what is possible in relation to implementing the new tool. The sessions were facilitated by an external consultant, and the researchers were present in the room as observers (Hammersley and Atkinson, 1995). Their role was to trace the systemic roots of specific and recurring problems and conceptualize these as inner contradictions in the past and present activity systems. This method was also chosen because research on implementation emphasizes the importance of involvement of participants as well as context-adapted implementation strategies as two important factors for successful implementation (Fixsen *et al.*, 2005).

Data from the field study, contradiction study and the barrier screening were used as mirror data in the workshop and in the analysis. Mirror data are defined as data representing the present state of work practices (Engeström *et al.*, 2012), and these data provide the practitioners with a mirror reflection of their activity by presenting examples of current practice.

The session was designed according to the following phases. First, participants were asked to describe and give an example of past practice before testing of the screening tool started, e.g. how the workflow performed for elderly medical patients. Second, participants had to describe their practice while testing the screening tool, e.g. which interventions were performed at the private residence of the at-risk medical elderly patients? Third, all groups made suggestions about how to implement the screening tool and redesign their work activity in the future in cooperation with the geriatric team, the two municipalities and the general practitioner. All phases were used to examine experiences and contradictions.

The workshop was conducted in a classroom in the hospital equipped with a whiteboard and a flipover chart. The whiteboard was used by the different groups to present their prepared data. The flipover chart was reserved for ideas and tools created collectively during the session. The participants were divided into four groups (Municipality B, Municipality K including the general practitioner, the geriatric team and the researchers). The sessions were videotaped and recorded on tape. The first author (JK) and two research assistants transcribed the recordings.

### 3.5 Data analyses

JK and JP performed the analysis using a step-by-step process. First, the analysis of the activity system began by holding up a mirror of the past object of the activity, the historical object, understood as the recurrent collective activities that the practitioners performed before the screening was tested. This analysis comprised both historical and actual empirical analysis of the past practice.

In this theoretical perspective, the new screening tool is a cultural mediator that is used to change the daily practice of the practitioners (Engeström, 1987). The transformation of the new activity system and the new object, the collective endeavors of the future activity and the resulting changes in the way the relationships among interactions within the activity system are mediated are analyzed.

In the process of analyzing the past and the new object, disturbances, ruptures and conflicts of underlying inner contradictions in the activity system are described. These contradictions create the dynamics of the transformation of the activity system. Finally, work-related interaction and collaboration, understood as the acting subjects’ relationships with the objects of work and with each other, are analyzed (Engeström, 2009, 2011).

The results from this workshop are presented in the next section and will be used to decide whether the screening tool is going to be implemented or not. If the tool is going to be implemented, the results will be used to plan and follow an implementation strategy.

## 4. Results

### 4.1 Historical perspectives on practice in the municipalities

The brief historical examination of past practices from the two municipalities analyzed as an activity system showed that allocation of visitation services to the citizens was decided by the Health Act and the Communication Agreement. One of the leaders from the municipalities reported:

We have lived in a world called services; visitation services.

(Leaders, Municipalities B, 2014)

These visitation services mediated the written and the oral communications. For example, the practitioners talked about what help the citizen needed from the nursing assistant (e.g. food or cleaning) and what nursing task the citizen needed. In an ideal practice, the written communication for the nursing tasks consists of a complete nursing assessment, but in practice, this was described as visitation services (e.g. pressure ulcer care for the next three days or administration of medication). Furthermore, at meetings, visitation services delivered by the nursing assistants were discussed first, then from the nurses. These priorities and articulations supported the focus on visitation services, which mediated that it was the practitioner with the least competence (the nursing assistant) who first visited the citizen after discharge. Each visitation service was allocated a fixed time, which did not lead to the use of clinical judgment but focused on the visitation service. Thus, the past objects for the collective activity were the visitation services for known citizens.

### 4.2 Contradictions of municipalities in the past activity system

**Figure 2:**
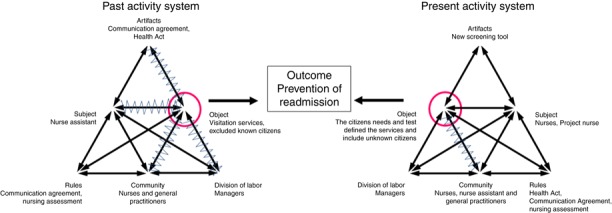
The municipality’s transformation of the activity system and its contradictions

The daily practice in the municipalities’ picture of an activity system created primary contradictions in the system (Figure 2). Contradictions are marked with an arrow. There was an implicit expectation from the nurses that the first practitioners who visited the citizen had a holistic view. The nurses expected that they had a more comprehensive holistic view than the nursing assistants. This expectation created a contradiction between the priorities of the person with the lowest competence (the nursing assistant) visiting the citizen first after discharge and the embedded expectations from the nurses. Nurses from both municipalities pointed out:

It was probably this [that it was the nursing assistant who first visited the citizens] that did not always succeed.

(Nurse, Municipality K, 2014)

Despite the managers’ responsibility, the past object (visitation services) led to blindness of the managers to send the right competency for the citizens. The managers’ motivation for fulfillment of the past object was to fulfill the requirements of the Health Act. The object was a political and society-created need mediated by the Health Act.

Another contradiction related to the past object arose because of the Communication Agreement, which states that practitioners are not allowed to send information about known citizens between the primary and secondary sectors without permission from the patients; but information is important for the communities to plan their visit to the citizens after discharge. Furthermore, the Communication Agreement, understood to be a tool, mediated a contradiction between the hospital and the municipalities for unknown citizens. The municipalities only received information about patients if they had sufficient functional decline. Thus, the agreement itself created primary contradictions of communication exchange.

### 4.3 The present activity system in the municipalities

Nurses from both municipalities articulated how the screening tool put focus on at-risk patients. When the citizen was categorized as at-risk or not, this mediated triage in terms of resources and, at Municipality K, it also mediated medical attention. Thus, the Communication Agreement and the Health Act floated in the background and categorization was put in the foreground. Categorization was used to simplify the complex elderly medical patients. A nurse commented:

You develop yourself to handle something unexpected […] “I do not know what this is about” but then you can work in a structured way to find out. That comes into focus.

(Nurse, Municipality B, 2014)

When a citizen was categorized as at risk, the categorization mediated that the nurses’ professional view was sharpened in the form of a more holistic view. This led to the active involvement of the citizen. When the services were not predefined, the nurses asked the citizen about their hospitalization and their needs. Also, both municipalities initiated the use of systematic tests to assess citizen needs. Moreover, all patients were screened by the geriatric team in the hospital; and about one quarter of the at-risk patients were not known to the municipalities. The present object for the new activity system became the citizens’ needs and tests that defined the services and included unknown citizens.

### 4.4 Contradictions of the municipalities in the present activity system

The only primary contradiction in the present activity system mentioned by the research group was that there was doubt created whether the nurses in Municipality K, where only one project nurse performed all the interventions, experienced ownership of the project. This design had an advantage in relation to systematic handling of the process, but some of the nurses perceived this as exclusion (Figure 2).

### 4.5 Transformation from the past to the present activity system

The new object mediated that there was a need for a more holistic view and therefore the nurses were the first to visit the citizen after discharge. Second, the category of at-risk citizens mediated that the nurses engaged with citizens who were in need of help without any specific assigned task. This was unlike the past activity system, where they engaged in assigned tasks from the Communication Agreement and the Health Act. Thus, the new object mediated movements in professional identity because the nurses had to decide themselves which tasks were the most important for the citizens. This also mediated that the nurses involved the citizen whereby the relationship between the nurses and the citizens came into focus.

Having to take responsibility for deciding which assignments were most important for the citizens also mediated a change in the relationship between the nurses and the nursing assistants and made the importance of the competencies of both professions visible to the leaders if the outcome, prevention of readmissions, was to be fulfilled:

We have learned the importance of supporting the nursing assistant. We cannot expect that they are the one to have a holistic view of at-risk citizens.

(Leader, Municipality B, 2014)

The new screening tool mediated pedagogical competencies because the nurses were more aware of the importance of support for the nursing assistant if the object and the outcome were to be fulfilled.

In Municipality K, the relational factor was also important in collaborations between the nurses and the general practitioners. This municipality chose to test at-risk citizens on their need for a general practitioner. The test was recommended by the Board of Health and the project nurses experienced that the use of the test improved competencies:

The test creates a systematic view in our approach to the citizens. We have become more aware of the entire citizens by using the test. The test has also helped to make our work more visible.

(Project nurse, Municipality K, 2014)

The visibility is evident in collaborations with the general practitioners. A general practitioner expressed:

I experience that the types of inquiry have changed. Before I received many strange inquiries from the nurses but now I receive many concrete inquiries.

(General practitioner, Municipality K, 2014)

This response shows that the use of the screening and the results from the test mediate a common language. The language was also mediated by a color code assigned to citizens after performing the test, which provided clarity about the need for medical competencies. The general practitioners express:

When the at-risk citizen is assigned a colour code, I know precisely that it is a citizen I have to take care of and act on to prevent readmission. Before, I did not get this specific information and thus there was more uncertainty about the effort I had to deliver.

(General practitioner, Municipality Y, 2014)

The use of the test mediated a change in workflow for the general practitioners. The concrete results from the test, including the color code, created a common focus on the outcome: preventing readmissions.

### 4.6 Historical perspectives on practice and the contradictions in the geriatric team

A historical examination of past practice and the contradiction in the geriatric team analyzed as an activity system showed that the team collectively carefully assessed admitted patients every morning. At interprofessional meetings, they decided which patients should be examined by the geriatric team. Essential competencies were comprehensive knowledge of geriatrics, a strong interdisciplinary focus and the use of advanced time- and resource-consuming screening tools, e.g. The De Morton Mobility Index (DEMMI) (de Morton *et al.*, 2008). Responsibilities were shared between the practitioners in the geriatric team. The practitioners were dependent on each other and personal relations created professionalism. The leader commented:

Often two practitioners attend the patient because then we can discuss what the right thing to do is and especially because we have different professions, we get a more holistic view of the patients.

(Leader, geriatric team, 2014)

The object for the activity system is an interdisciplinary holistic view for selected patients.

**Figure 3:**
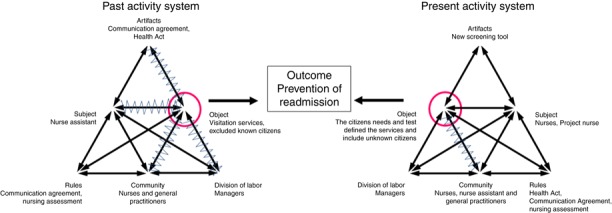
The geriatric team’s transformation of the activity system and its contradictions

The only structural contradiction created in the activity system was in form of the nurses in the geriatric team who also had to go on shift in the ED (Figure 3). The consequence was that several days a week, the geriatric team was not represented with a nurse and nursing competency, which become a contradiction in relation to the strong interdisciplinary focus.

### 4.7 The present activity system in the geriatric team

The geriatric team experienced that the screening mediated a more interdisciplinary collaboration than before, e.g. the doctors performed a physiotherapy task. Thus, the screening mediated new competencies and mediated increased collaboration between the team members because they had to discuss the results of the test with each other. In addition, the geriatric competencies were assessed with special significance to interpreting the results of the screening.

The screening mediated change in the geriatric workflow; the geriatric nurse stopped weekend duty in the ED and worked full time in the geriatric team. Furthermore, the screening mediated security in relation to collaboration with the municipalities because they knew that someone was to follow up upon discharge, which they did not know before. A nurse expressed:

It creates a big safety to know that there is somebody to take care of the patients when they are discharged from the hospital.

(Nurse, geriatric team, 2014)

The geriatric team experienced that the screening helped them to see more patients than before. The new object was an interdisciplinary view of many patients.

### 4.8 Contradictions of the geriatric team in the present activity system

The new object created some contradictions (Figure 3). The first contradiction was that the tool created a primary contradiction in relation to time. The geriatric team had to spend a lot of time on tasks they perceived unrelated to their perceived core task, e.g. finding bathrobes. Second, the screening created secondary contradictions compared with more advanced tests, e.g. DEMMI. The DEMMI test had to wait to be implemented because of the new screening tool, which created frustration among the physiotherapists, who saw the DEMMI test as more relevant for physiotherapists.

The geriatric team found that the screening created primary contradiction in choosing the right patients, understood to be those patients who the team assessed as most relevant from a geriatric professional view, built on many years of experience. Some of the patients who were screened were “in too good condition” and therefore the team found that too many patients were screened unnecessarily (Figure 3).

### 4.9 Transformation from the past to the present activity system

The most central results in the geriatric team from the past to the present activity systems were all the contradictions that the new screening created. Despite these contradictions, the screening also mediated a transformation of the activity system for the geriatric team, whereby the new object became an interdisciplinary view of many patients, rather than before when the object was an interdisciplinary view of a few selected patients. The transformation put the competencies of the geriatric team in the foreground. A physiotherapist from the geriatric team expressed:

Before the project, we thought that anybody could handle the screening, even a medical student. But now we have learned that our special geriatric knowledge is extremely important to understand the results.

(Physiotherapist, geriatric team, 2014)

As a result of this transformation of the object, the competencies of the geriatric team changed and their awareness of these special competencies came into focus as especially significant when interpreting the results of the screening. Furthermore, the screening mediated a structural change for the nurse in the team, which gave the nurse the opportunity to work every day in the geriatric team instead of working shifts in the ED.

### 4.10 Development of cross-sectoral collaboration

Before the Info-65 project, both the municipalities and the geriatric team were following their scripted roles, each concentrating on the successful performance of the assigned actions. A participant from the geriatric team explained:

Before the project began, we started at 8 am reading OPUS notes [OPUS is an electronic patient record]. Reading OPUS notes means reading about patients who are admitted, blood pressure and other vital signs. We also went through the files in order to depict which patients we immediately found relevant for our team to review.

(Participant, geriatric team)

Or a nurse from the municipalities stated:

It was always the nursing assistant who visited the citizens first after discharge.

(Nurse, Municipality B)

These communicative examples show how the script was coded in rules and plans or imbedded in tacitly assumed traditions. The script coordinated the participants’ actions as if from behind their backs, without being questioned or discussed. Even though the three groups have a common outcome, their focus was on their own entities and not the entities of the others.

Through the Info-65 project and in the mirror data, it has been highlighted that if the issue of readmission of elderly medical patients is to be prevented, cooperation between the primary and secondary sectors is required. This can be achieved by developing a new collective understanding of the commonly shared object of the activity and new tools to achieve a collective outcome. The new screening tools mediate some signs of a more collectively shared cooperation. A nurse expressed:

The fact that we have had this intervention, that we have tested our citizens, has increased our cooperation with the general practitioners. It means that we collectively have been able to make a plan and support what needs to be done for the citizen.

(Nurse, Municipality K)

and:

It has created confidence to know that the citizens are examined by a nurse after discharge. It is a huge advantage from previously, particularly for the unknown citizens. Now we complement each other and maybe this could prevent readmission.

(Participant, geriatric team)

These communicative expressions shown how the screening tool and the intervention in the municipalities were collectively viewed as an acceptable way to conceptualize and solve the common problem with elderly medical patients. The screening tool has mediated a transformation from the focus of each performing its own tasks and roles to a focus on solving a common problem. The participants go beyond the confines of the given script but they still do not explicitly question or reconceptualize the script.

Data are not consistent in relation to whether all groups have transformed their own organization and interactions in relation to the shared object. On the one hand, the municipalities have transformed their organizations in that they have reorganized who is supposed to visit the citizens first, the priority is the highest competency, and at the same time they have strengthened communication with the general practitioners. The geriatric team has made changes to roles and tasks in daily practice. This could indicate that the new screening tool has mediated a reconceptualization of both the object and the script and thereby developed a general structure of communication and cross-continuum collaboration. On the other hand, when discussing the future practice of the new screening tool, statements like the following emerged:

Re-screening, it is a very dangerous issue. We do not have the resources to re-screen. […] Why should it be a task within the geriatric function? It might as well be included among the tasks of the ED nurses when they receive the patients.

(Participants, geriatric team)

When talking about implementation of the new screening tool in all medical departments, participants from the geriatric team expressed:

Who should implement the tool? Do not count on us in the geriatric team. Now we have to implement the DEMMI test, which has had to wait due to this project.

(Participants, geriatric team)

Statements like these from the geriatric team, e.g. suggesting that the ED nurses should take responsibility for the screening tool, were made even though they were aware of the mirror data that identified that the nurses would not implement and use the tool. This was because the new tool did not support the flow, and the contradictions identified in the analyses of the present activity system for the geriatric team indicated that there was no cross-continuum collaboration. Instead, the contradictions and the statements indicate how the geriatric team tries to stabilize their object (historical object), tools and scripts; that is why the new screening tool created so many contradictions during the project. Instead, the analyses identify that the geriatric team developed a form of a temporal object and pseudo-cooperation during the project, as indicated by the existence of a latent competing script that became a contradiction for cross-continuum collaboration and for the implementation of the new tool.

## 5. Discussion

This study shows that testing a new screening tool created different transformations both in the municipalities and in the geriatric team in the ED. These transformations raise the question whether sustained cross-continuum collaboration between the two sectors was developed and to what extent it is possible to implement the new tool. Exploring the particular past and present object of activity revealed the relational nature of activity systems, involving primary nurses, the geriatric team from the ED and the general practitioners, working together in “knots” (Engeström, 2008), meaning that all the participants in the project collectively tried to redesign their practice to reach the collectively shared outcome; to prevent readmission of older medical patients. The collaboration was, especially for the municipalities, not considered rigid, with predetermined rules or a fixed central authority. Instead they could design and organize the intervention for at-risk patients as they believed would make the best sense for the citizen, which is a motivational factor to adapt to local context and to implement the new tool (Craig *et al.*, 2008). This categorization of at-risk patients, mediated by the screening tool, established a new collective activity whereby the nurses provided more comprehensive care than before the implementation of the tool. This categorization actually included the citizens in the discourse, which is in contrast to other studies from Engeström (2000), where the object, e.g. the patient, was excluded from the discourse. The categorization mediated the transformation from coordination to cooperation, internally in the municipalities, between the nurses and nursing assistants, and between the nurses and the general practitioners.

Before the project, the practice of the municipalities was characterized by a number of primary contradictions in relation to preventing readmission of older medical patients, which is also demonstrated by other studies (Marcantonio *et al.*, 1999; Parke *et al.*, 2011). The contradictions also included coordination with the geriatric team, e.g. the municipalities were not informed about unknown citizens. The new screening tool mediated a new collective object and transformation of the activity system. This transformation mediated that the nurses and their leaders experienced far less contradictions in their practice. Only one factor was questioned in one of the municipalities: the choice of one key person or opinion leader who was responsible for the interventions with the older medical patients after discharge. The implementation literature indicates that there is some evidence that opinion leaders (Flodgren *et al.*, 2011) or key persons promote evidence-based practice and improve patient outcomes. Even the project nurse in our study was considered likeable, trustworthy and influential (factors that define an opinion leader) by colleagues. The decision to choose one person created contradictions in the present activity system, because many of the other nurses did not experience ownership of the project and some nurses had the experience of exclusion. The possibility of transforming the scripted flow from coordination, where the nurses focus on their respective individual object, to communication, where the nurses reformulate their role and the script of their interaction in relation to a sustained shared object (Engeström, 2008), did not occur. In a possible future implementation of the screening tool, the choice of only one key person will create a secondary contradiction in the activity system. The implication is that the implementation plan must include several implementation strategies if the implementation is going to be a success (Powell *et al.*, 2012, 2015).

The implementation of the new tool created another transformation process in the geriatric team. Before the project, only one structural contradiction was embedded in the activity system, but the implementation of the screening tool created a tertiary contradiction internally in the geriatric team and a quaternary contradiction between the geriatric team and the municipalities. The geriatric team’s attempt to stabilize their historical object could be seen as a result of team history that is embedded in the history of the organization and in the history of the profession (Engeström, 2008). The mirror data describe what happened when the geriatric department was closed and the geriatric team was affiliated to the ED instead. The use of a comprehensive and sophisticated functional test such as the DEMMI was a sign of the geriatric team’s professional identity. Unlike the municipalities, the collaboration was formed with a predetermined role in form of an artifact – the new screening tool. This tool was perceived as simplified and could not replace the comprehensive screening tool as a sign of their professional identity. Even though the geriatric team was motivated to participate in testing and implementing the tool and their leader was characterized as an early adopter (Rogers, 1983), our results identified how this collaboration created a pseudo-cooperation and a temporal object. The results tend to be categorized according to Fraser’s (2007) definition of pilotitis, which is understood as dissatisfaction with isolated pilot projects that may have been successful but were not rolled out into enduring changes. Our results show that it is possible to develop collaboration between two sectors in the testing or pilot period and overcome some of the historic fragmentation of health care service delivery between cross-continuum collaborations (Loehrer *et al.*, 2015). This is shown by transformation to a shared object and sidebars in the form of a change in collaboration, internal in the municipalities and in the geriatric team in the form of changes in roles and tasks in daily practice. But the results also showed that the new screening tool has not mediated a general and sustained transformation in the cross-continuum collaboration and has not mediated a transformation to a collective sustained script. Some implementation research would interpret these results as if the implementation and dissemination went wrong (Wieringa and Greenhalgh, 2015). In contrast, our results add to the knowledge that implementation of screening tools always mediates different contradictions and transformations in different sectors. Our results indicate that we need to adapt to local contexts if a new screening tool is to be implemented. One limitation against this adaptation is that the nursing assistants were not part of the study even though they were the first to visit the citizens in the past activity in the municipalities. Their perspectives could have qualified the contradictions and the transformations of the past and future activities in relation to the collaborations with the nurses.

## 6. Conclusion

This study has shown that implementation of screening tools always mediates different contradictions and transformations in different sectors. Screening tools are not objective, neutral or “acontexual” artifacts and must always be adapted to the local context. CHAT offers a way to understand the leading collective object when working with organizational transformations and implementation and focus not only on the structural changes but also the cultural aspects of organizational changes if we want to reach a sustained change and implementation of the new screening tool in different sectors.
